# Active smoking and exposure to secondhand smoke and their relationship to depressive symptoms in the Korea national health and nutrition examination survey (KNHANES)

**DOI:** 10.1186/s12889-015-2402-1

**Published:** 2015-10-14

**Authors:** Sun Jae Jung, Aesun Shin, Daehee Kang

**Affiliations:** Department of Biomedical Science, Seoul National University College of Medicine, Seoul, South Korea; Department of Preventive Medicine, Seoul National University College of Medicine, Seoul, South Korea; Cancer Research Institute, Seoul National University Hospital, Seoul, South Korea

**Keywords:** Cigarette smoking, Secondhand smoke, Depressive symptoms

## Abstract

**Background:**

The relationship between tobacco smoking, including secondhand smoking, and depression has been assessed. The purpose of this study was to evaluate the association between secondhand smoking among current, former and never smokers and depressive symptoms. For secondhand smoking, gender differences and sources of exposure were examined.

**Methods:**

Data from 34,693 participants from the fourth and fifth Korean Health and Nutritional Examination Survey (2007–2012) were analyzed in 2014. Self-reported exposure to active (current, former or never) and secondhand smoking and depressive symptoms experienced during the past year were analyzed using logistic regression. The dose–response relationship between duration of secondhand smoke exposure and depression was assessed with stratification by gender and sources of exposure (at home only, at the workplace only or both).

**Results:**

Regardless of their smoking status, all women who had secondhand smoke exposure at home reported more depressive symptoms than non-smoking women without any exposure to secondhand cigarette smoking (OR 1.43, 95 % CI 1.04–1.96 for current smokers; OR 2.32, 95 % CI 1.04–5.16 for former smokers; OR 1.25, 95 % CI 1.08–1.43 for never smokers). There was also a significant dose–response pattern (*p*-trend <0.001) for the duration of secondhand smoke exposure at home among women. No significant association was found between smoking and depressive symptoms in men.

**Conclusions:**

There was a significant association between secondhand smoke exposure at home and depressive symptoms in women. Secondhand smoke exposure at home was associated with depressive symptoms in a dose–response manner.

**Electronic supplementary material:**

The online version of this article (doi:10.1186/s12889-015-2402-1) contains supplementary material, which is available to authorized users.

## Background

Smoking tobacco is well known to be harmful to overall health and is a major cause of death and disease. In Korea, 30.8 % and 5.7 % of deaths among men and women, respectively, have been attributed to active smoking [1]. According to one study, 8,284 people died of either lung cancer or ischemic heart disease due to current smoking and an additional 422 non-smokers died of the same diseases due to secondhand smoke (SHS) in 2010 [2]. Furthermore, approximately 100 billion cigarettes are sold annually in Korea; thus, according to World Health Organization (WHO) data, Korea is the 8th largest cigarette market [3]. The smoking rate among men and women was approximately 40 % and 5 %, respectively, in 2011 [4]. Similar to other countries [5], several public policies have been established to protect the general population from SHS in Korea since 1995 [6]. The policies mainly focus on exposure in open public places such as workplaces, large theaters, hospitals, schools, auditoria, gymnasia and public transportation. These policies successfully led to a decrease in the prevalence of smoking among men (46.7 % in 2007 and 44.9 % in 2010) [7]. However, the prevalence of smoking among women increased during the same period [8]. In 2011, approximately 55.2 % of men and 37.2 % of women who participated in the Korean National Health and Nutritional Examination Survey (KNHANES) reported that they were exposed to indoor workplace SHS, whereas 16.7 % of men and 4.9 % of women were exposed to SHS at home [9]. Recently, there has been a growing number of debates on banning smoking inside apartments in Korea. Furthermore, there is also increased awareness of the positive effects of smoking bans at home [10]. Studies have shown inconsistent results concerning the potential factors associated with SHS exposure [11, 12].

While the harmful effects of smoking have frequently been discussed in relation to overall mortality of other diseases such as cancer, some studies have also begun to focus on the adverse effects of active smoking on mental health, presenting the harmful effect of active smoking on depression [13, 14]. Nevertheless, the results regarding SHS and depression are controversial [15, 16].

In Korea, the prevalence of depression, measured using the Center for Epidemiological Studies-Depression Scale (CES-D), was 23.1 % and 27.4 % among men and women, respectively [17]. Koreans who immigrated to the U.S. showed approximately 30.0 % to 36.0 % prevalence of depression, as measured by the CES-D [18, 19]; these rates were relatively high compared to the general American population (3–21 %) [20–22].

Many studies have evaluated the bidirectional relationship between current smoking and depressive symptoms, but fewer studies have examined the association between SHS and depression. One study on Japanese workers showed a positive association between SHS exposure and prevalence of depression [23]. Depression is twice as prevalent in females [24], and there may be different etiologies for depression in men and women. In addition, it has been reported that gender significantly modifies the association between smoking and depression; the prevalence of depression tends to be higher among female current smokers compared to female former or never smokers, whereas the prevalence among male current smokers and never smokers does not differ [25]. In addition, among non-smokers, women are more frequently exposed to SHS at home than men [9, 26]. The source of SHS exposure should also be distinguished to assess the different association between SHS and depression. Another study conducted in Korea assessed the association between SHS and depressive symptoms in middle and high school students using a school-based survey. This study showed a dose-dependent association between SHS and depression [15, 27].

The purpose of the current study was to explore the association of smoking status (current, former or never) and SHS exposure with depressive symptoms and to assess the possible dose–response relationship between SHS exposure duration and depressive symptoms stratified by gender using information from a nationally representative survey in Korea.

## Methods

### Study participants

**Fig. 1:**
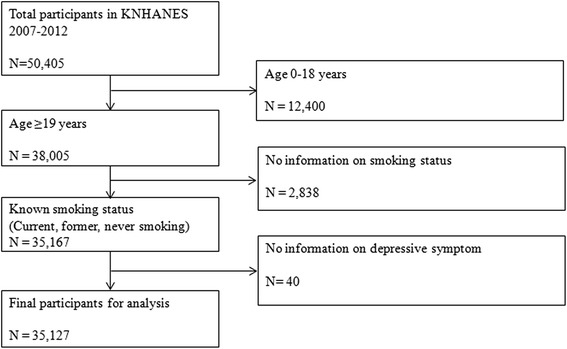
Data management flow (KNHANES 2007–2012)

Data were drawn from the fourth and fifth Korean Health and Nutritional Examination Survey IV-V (KNHANES 2007–2012). KHNANES provides nationally representative data by selecting participants from the non-institutional population in South Korea using a complex stratified multistage probability-clustered sampling design [28]. Since 2007, surveys have been conducted annually using in-person interviews and a multi-stage clustered probability design. More information about the sampled participants can be found elsewhere [28]. From a total of 50,405 participants in KNHANES 2007–2012, data from 34,693 respondents over the age of 18 years and without missing information on smoking and symptoms of depressive and stress were used in the final analysis (Fig. 1).

### Measures

All the interviews regarding socioeconomic factors and self-perceived health status were conducted by trained interviewers using structured questionnaires. Smoking status, alcohol consumption, perceived stress and depressive symptoms were all self-reported. To ascertain smoking status, participants were asked the following questions: “How many cigarettes have you smoked in total?” and “Do you currently smoke?”. If the participants answered that they have smoked 100 cigarettes or more in their lifetime and that they smoke currently, there were categorized as “current smokers”. If participants answered that they do not smoke currently but that they had smoked 100 cigarettes or more, they were classified as “former smokers”. People were labeled as “never smokers” if they reported that they have smoked less than 100 cigarettes in the lifetime. Those who answered that they either smoked every day or occasionally were additionally asked how many cigarettes they consumed per day or how many days they smoked in the previous month. To assess the presence and the amount of workplace SHS exposure, all participants were asked, “How many hours per day are you exposed to other people’s cigarette smoke in the workplace?” Participants were also asked if there is any other family member who smokes inside the house everyday other than themselves. If the participants answered “yes”, they were asked the number of smokers other than themselves and the total hours per day of exposure to cigarette smoke. For both exposures, participants reported how many hours they were exposed by checking “no exposure”, “exposed <1 h/day” and “exposed ≥1 h/day”. Depressive symptoms were assessed with the following question: “In the past year, have you felt extremely sorrowful or despair for more than 2 weeks?”. The participants answered this question as “yes” or “no”. This questionnaire was used to identify people with depressive mood from the beginning of KNHANES (1998). It is widely used to measure participants with clinical or subclinical depressive symptoms, which are expected to be a proxy for depressive disorder [29, 30]. In addition, people were asked whether they ever had a diagnosis of clinical depression confirmed by physicians. Participants who answered “yes” had to report the age at their first diagnosis of depression. The participants’ level of stress was asked by the following question: “During ordinary days, how much do you feel stressed?: very much, moderate, somewhat or not at all.” To measure employment status, participants were asked about their current financial activity. Excluding the people who answered that they are not financially active or they are unemployed, have no intention to work or do not have the ability to work, participants were asked which of the following employment types applied to them: wage employee, independent business owner or worker in the family business. The Alcohol Use Disorders Identification Test (AUDIT) scores were classified following the conventional risk level cut-off (–7/8–15/16–19/20–40) [31]. Self-rated health was measured by asking the participants the following question: “What do you think about your current health status: very good, good, normal, bad or very bad?”.

### Statistical analysis

As data from KNHANES were derived from multistage complex probability sampling to represent the entire South Korean population, population weightings were applied in the analyses. The PROC SURVEY procedure in SAS 9.3 (SAS Institute Inc., Cary, NC, USA) was used to apply stratification, primary sampling units and population weights. Because the data were integrated from 2007 to 2012, we calculated the separated integrating weight for each year in proportion to that year’s response rate. Continuous variables were categorized by order. The exposure categories included four SHS statuses (Never exposed to SHS, SHS exposure at home only, SHS exposure at workplace only and SHS exposure in both places) and active smoking (Current smoker, former smoker and never smoker). Never smokers who answered that they were not to exposed to any SHS served as the reference groups for both genders. Age was grouped as 19–39 years, 40–59 years and 60 years and older. Body mass index (BMI) was categorized following the WHO criteria for Asians [32]. The participants selected for analysis were those who did not have missing values on the main exposure (smoking status) and outcome variables (depressive symptoms), and the missing values on other variables were coded separately and included in the models to calculate odds ratios. To describe the distribution of baseline characteristics, the PROC SURVEYFREQ procedure was applied. The likelihood ratio test was conducted to evaluate possible covariates in the model. Each possible covariate was added to the model, and −2 Log likelihood was compared with previous model. If the difference was statistically significant in *χ*^2^-distribution (degree of freedom = 1, *p*-value <0.05), the variable was selected as an adjustment variable. Age, marital status, education, employment status, self-reported stress, self-rated health and alcohol consumption (measured as the AUDIT score) and past diagnosis of depression by physicians were selected as the final covariates in the model. After stratifying for gender, adjusted odds ratio for depression were calculated by 12 categories (3 × 4) regarding 3 groups for smoking status (current, former, never smoker) and 4 groups for exposure to SHS (No exposure to SHS, SHS at home only, SHS at workplace only, SHS at both home and workplace) using PROC SURVEYLOGISTIC. For those who had never smoked, SHS exposure in the workplace and at home was grouped by dose of exposure (no exposure, <1 or ≥1 h/day). Trend tests were performed by using the order of categories. Additionally, as participants had information concerning whether they had been diagnosed with clinical depression by physicians, we conducted a sensitivity analysis to avoid selection bias by excluding the participants who reported a history of depression diagnosed by physicians. All statistical analysis were conducted with SAS 9.3; the significance level was set at *p* <0.05. All analyses were conducted in 2014.

### Ethics

All KNHANES participants provided written consent to participate in the survey and for their personal data to be used. This study, which conducted analyses with publicly open data, was approved by the Institutional Review Board of the Seoul National University Hospital, Korea (IRB No. 1408–068–602).

## Results

### Baseline characteristics of the study population

As shown in Table 1, there was a total of 35,127 participants in this study, of whom 35.7 % were current smokers, 10.8 % were former smokers and 53.5 % had never smoked. Among the 14,886 men, the weighted percentages of current and former smokers and those who never smoked were 62.0 %, 18.2 % and 19.8 %, respectively. Among the 20,241 women, the weighted percentages of current and former smokers and those who never smoked were 10.2 %, 3.6 % and 86.3 %, respectively. The weighted percentages of people who had any exposure to SHS were 53.4 % among men and 42.7 % among women. Among the men, current smokers were slightly older than those who had never smoked (14.4 % compared to 13.5 % were aged ≥60 years), but among the women, current smokers were younger than those who had never smoked (57.5 % compared to 36.6 % were aged 19–39 years). Among never smokers, a greater percentage of the men attained education for 12 years or more compared to women (40.7 % of men and 27.4 % of women attained ≥12 years of education). Women who had never smoked were less likely to report no financial activity compared to current female smokers (never smoked, 51.0 %, compared to current smoker, 52.6 %), whereas men with no financial activity were more likely to have never smoked (29.3 % compared to 20.5 % of current smokers). Women who had never smoked had a lower BMI than those who smoked. There was a greater proportion of both male and female smokers than non-smokers among the participants who reported alcohol consumption (“ever drank”) (97.5 % of men and 92.9 % of women) and higher self-reported stress among current smokers compared to those who never smoked. Similarly, participants who never smoked reported better health status than smokers (Data not shown). A total of 1,468 (9.9 %) men and 3,658 (18.1 %) women reported depressive symptoms.

**Table 1:** Baseline characteristics of participants by depression status in KNHANES 2007−2012

(*N*=, weighted N=)						
	Men (*N* = 14,886, weighted *N* = 18,449,986)			Women (*N* = 20,241, weighted *N* = 18,929,457)		
	depression (−) (*N* = 13,418) (Weighted *N* = 16,715,177)	depression (+) (*N* = 1,468) (Weighted *N* = 1,734,808)	*P*-value	depression (−) (*N* = 16,583) (Weighted *N* = 15,575,753)	depression (+) (*N* = 3,658) (Weighted *N* = 3,353,704)	*P*-value
General factors	Weighted %	Weighted %		Weighted %	Weighted %	
Smoking status			0.015			<0.001
Current smoker						
Exposed to SHS	34.9	37.8		4.9	9.1	
Not Exposed to SHS	26.6	29.8		4.2	6.4	
Former smoker						
Exposed to SHS	8.4	7.6		1.3	2.1	
Not Exposed to SHS	10.0	8.7		2	2.3	
Never smoker						
Exposed to SHS	9.6	8.0		31.5	31.5	
Not Exposed to SHS	10.6	8.1		56.1	48.6	
Age			<0.001			<0.001
19−39	43.7	36.2		41.0	33.0	
40−59	39.8	43.3		38.5	39.2	
≥60	16.6	20.5		20.5	27.8	
Marital status			<0.001			<0.001
Never married	25.7	25.5		17.5	15.0	
Married-living with partner	70.3	64.4		67.7	61.0	
Married-others	4.0	10.1		14.8	24.0	
Education attainment			<0.001			<0.001
<6 years	12.0	19.7		24.1	35.4	
6−8 years	10.0	13.4		9.8	11.7	
9−11 years	42.2	41.2		37.7	33.5	
≥12 years	35.9	25.7		28.5	19.4	
Employment status			<0.001			0.002
Wage working	49.4	38.3		32.4	28.7	
Self-supporting	26.9	29.1		11.1	10.8	
House-employed	1.6	2.5		5.9	5.8	
No financial activity	22.0	30.0		20.6	54.6	
Number of family members			<0.001			<0.001
Living alone	4.9	7.7		6.2	9.3	
Living with one family member	19.4	20.5		19.2	22.9	
Living with two family members	26.8	26.8		24.6	25.9	
Living with ≥3 family members	48.8	45.0		49.9	41.9	
Body mass index (kg/m2)			0.5			0.018
<18.5	3.0	3.5		6.7	6.5	
18.5−22.9	34.9	36.1		45.1	42.7	
23−24.9	25.6	26.1		20.8	20.8	
25−29.9	32.2	31.1		23.2	24.5	
≥30	4.2	3.2		4.2	5.5	
Alcohol dependence by AUDIT score			<0.001			<0.001
0−7 (Normal)	44.2	39.6		84.1	76.1	
8−15 (Mild dependence)	34.2	26.7		12.6	15.6	
16−19 (Moderate dependence)	11.2	12.9		1.6	3.8	
20−40 (Severe dependence)	10.5	20.8		1.7	4.5	
Self-reported stress			<0.001			<0.001
Very high	2.7	16.4		3.0	17.5	
High	19.5	42.0		21.5	44.7	
Moderate	61.7	37.5		60.4	32.7	
Little	16.0	4.2		15.1	5.0	
Self-reported health status			<0.001			<0.001
Very good	5.9	3.8		4.0	2.4	
Good	37.3	21.5		31.8	19.3	
Normal	43.8	41.4		44.8	38.6	
Bad	11.7	27.2		16.7	30.4	
Very bad	1.3	6.1		2.7	9.3	

### Association between smoking and depressive symptoms

In the multivariate analyses, stratified by gender (Table 2), there was no significant association between smoking and depression in men. However, women who answered that they were exposed to SHS only at home showed significantly increased ORs on depression regardless of their active smoking status (Current, former or never smokers): Odds ratio (OR) 1.43, 95 % CI 1.04–1.96 for current smokers, OR 2.32, 95 % CI 1.04–5.16 for former smokers and OR 1.25, 95 % CI 1.08–1.43 for never smokers. In addition, among women, current smoker status combined with exposure to SHS both at home and workplace was marginally associated with elevated depressive symptoms (OR 1.57, 95 % CI 0.96–2.55). Likewise, for women who had never smoked, SHS exposure both at home and the workplace increased their OR of depressive symptoms by 12 % (OR 1.22, 95 % CI 0.99–1.404). In contrast, SHS exposure at the workplace did not show statistically significant associations with the likelihood of elevated depressive symptoms for any smoking groups.

**Table 2:** Association between smoking status (current, former and secondhand smoke (SHS)) and depressive symptoms

Smoking status	Depression		
	No	Yes	OR^a^ (95 % CI)
	*N* (Weighted %)	*N* (Weighted %)	
Men			
Smoking status			
Current			
No exposure to SHS	3,859 (26.6)	470 (29.8)	1.15 (0.86–1.55)
SHS at home only	526 (3.7)	85 (6.5)	1.42 (0.92–2.18)
SHS at workplace only	3,120 (26.2)	339 (25.4)	1.13 (0.83–1.54)
SHS at both home and workplace	525 (5.0)	75 (6.0)	1.03 (0.69–1.55)
Former			
No exposure to SHS	1,752 (10.0)	156 (8.7)	1.01 (0.72–1.41)
SHS at home only	68 (0.5)	14 (0.7)	1.27 (0.56–2.88)
SHS at workplace only	907 (7.3)	84 (6.5)	1.17 (0.79–1.71)
SHS at both home and workplace	51 (0.5)	3 (0.3)	0.59 (0.16–2.16)
Never			
No exposure to SHS	1,409 (10.6)	123 (8.1)	1.00 (ref)
SHS at home only	102 (1.0)	10 (1.0)	1.10 (0.46–2.65)
SHS at workplace only	889 (7.6)	85 (5.7)	1.12 (0.76–1.64)
SHS at both home and workplace	96 (1.0)	11 (1.3)	2.05 (0.83–5.10)
Women			
Smoking status			
Current			
No exposure to SHS	630 (4.2)	217 (6.4)	1.25 (1.00–1.56)
SHS at home only	283 (2.0)	131 (4.4)	1.43 (1.04–1.96)
SHS at workplace only	214 (1.7)	68 (2.4)	1.26 (0.87–1.83)
SHS at both home and workplace	142 (1.1)	56 (2.2)	1.57 (0.96–2.55)
Former			
No exposure to SHS	283 (2.0)	70 (2.3)	1.04 (0.72–1.51)
SHS at home only	50 (0.4)	17 (0.9)	2.32 (1.04–5.16)
SHS at workplace only	71 (0.6)	22 (1.0)	1.60 (0.82–3.13)
SHS at both home and workplace	22 (0.2)	6 (0.3)	1.73 (0.53–5.62)
Never			
No exposure to SHS	9,932 (56.4)	1,985 (48.8)	1.00 (ref)
SHS at home only	1,782 (12.0)	459 (14.4)	1.25 (1.08–1.43)
SHS at workplace only	1,993 (13.3)	373 (11.0)	0.99 (0.84–1.17)
SHS at both home and workplace	805 (6.0)	191 (5.9)	1.12 (0.99–1.40)

^a^Adjusted for age, marital status, education, employment status, self-reported stress, self-rated health, alcohol dependence (AUDIT) and past diagnosis of depression by physicians

### Dose–response relationship between SHS and depressive symptoms

Among men who had never smoked and had occupational exposure to SHS, exposure for ≥1 h/day was associated with marginally increased odds of depression (OR 1.23, 95 % CI 0.97–1.54). Nevertheless, the test for a linear trend between SHS and depressive symptoms was not significant. The OR pattern was similar among non-smoking men who had only been exposed to SHS at home; however, none of the results reached statistical significance. In contrast, the results for women differed. Among non-smoking women, SHS exposure of ≥1 h/day both in the workplace and at home was significantly associated with increased odds of depressive symptoms (workplace: OR 1.32, 95 % CI 1.06−1.64; home: OR 1.71, 95 % CI 1.34−2.18). A longer duration of daily SHS exposure at home increased ORs (*p*-trend for home exposure, <0.001) (Table 3).

**Table 3:** Association between secondhand smoking and depression with different exposure duration

Characteristics	Male		Female	
	Case *N* (weighted %)	OR^a^ (95 % CI)	Case *N* (weighted %)	OR^a^ (95 % CI)
Workplace exposure (*N* = men 14,766, women 19,804)				
No exposure	859 (54.8)	1.00 (ref)	2,879 (77.2)	1.00 (ref)
<1 hour/day	389 (29.4)	0.92 (0.77–1.09)	470 (14.0)	0.89 (0.76–1.04)
≥1 hour/day	208 (15.8)	1.23 (0.97–1.54)	246 (8.8)	1.32 (1.06–1.64)
	*p*-trend	0.256	*p*-trend	0.159
Home exposure (*N* = men 14,846, women 20,194)				
No exposure	1,359 (91.5)	1.00 (ref)	2,896 (75.4)	1.00 (ref)
<1 hour/day	81 (6.6)	0.90 (0.65–1.23)	2,083 (17.1)	1.18 (1.02–1.35)
≥1 hour/day	24 (2.0)	1.21 (0.69–2.13)	215 (7.5)	1.71 (1.34–2.18)
	*p*-trend	0.977	*p*-trend	<0.001

^a^Adjusted for age, marital status, education, employment status, self-reported stress, self-rated health, alcohol dependence (AUDIT) and past diagnosis of depression by physicians

### Sensitivity analysis

In our data, 246 men and 1,124 women reported having a prior diagnosis of clinical depression confirmed by physicians. We excluded these participants and re-calculated the odds ratios for the association between each smoking status and depressive symptoms. Similar to the prior analysis including the participants with prior depression, only women showed a significantly increased odds ratio on depression with SHS at home only (OR 1.53, 95 % CI 1.09−2.15 for current smokers, OR 1.21, 95 % CI 1.04−1.40 for never smokers). Female former smokers who were exposed to SHS at home showed marginally increased OR on depression (OR 2.23, 95 % CI 0.95−5.28).

Likewise, we re-conducted the analysis using different exposure doses in workplace and at home while excluding participants with prior depression. Overall, results of the original data and the data with exclusion were very similar. There was no significant result among men. In contrast, women who were exposed to SHS in the workplace or at the home had significantly greater odds of depressive symptoms than those with less exposure (<1 h per day) (OR 1.38, 95 % CI 1.10−1.73 for women who had SHS exposure at the workplace and OR 1.69, 95 % CI 1.31−2.17 for women who had SHS exposure at home). Women who had SHS exposure at home showed a significant trend (*p*-trend <0.001) (data not shown).

## Discussion

This study evaluated the association between adults’ SHS and depressive symptoms by the exposure source in a nationally representative dataset. In this study, we found a significant association between active or secondhand smoking and depressive symptoms in women. Women who were exposed to SHS at home showed a stronger dose–response relationship than those exposed at the workplace. It is interesting that only women showed a significant trend in the dose–response relationship between SHS duration and depressive symptoms at home.

The association between SHS and depressive symptoms has been assessed in several studies [23, 27, 33–35]. In a study conducted with non-smoking Korean adolescents, exposure to SHS increased the likelihood of depressive symptoms in both genders after adjustment [33]. However, our study showed a significant OR only for women. Several other studies support our finding that women are more susceptible to SHS related factors [36] and associations between smoking and depression were more prominent in women [37]. This may be due to men’s higher rate of smoking, which contributes to women’s greater exposure to SHS at home [38, 39]. In our study, the most notable association was found for women who were exposed to SHS at home; there was a significant trend in the dose–response relationship between SHS and depressive symptoms compared to the SHS exposure at workplace. Another possible explanation is that many people are exposed to SHS, especially in more stressful job or home environments. In our data, women who reported severe stress levels were more likely to be exposed to SHS at both home and the workplace. In addition, women who were current or never smokers and exposed to SHS at home were more likely to report severe stress levels (Additional file 1: Table S1). Because stress is a well-known cause of depression [40], SHS is also linked with depression. One study showed that there are correlations between banning smoking in the home and lowering the risk of depression [41].

In a meta-analysis of 85 cross-sectional studies [42], a significantly increased prevalence of depression was found in current smokers compared to those who had never smoked (OR 1.50, 95 % CI 1.39–1.60). The magnitude of the increase in OR is very similar to our result. One study conducted in Korea showed a two-fold increased risk of depression in women who smoked compared to non-smoking women (OR 2.07, 95 % CI 1.51–2.83) [43]. In addition, a community health study in Korea suggested that smoking is particularly associated with depression in women [35]. Nevertheless, there are some contradicting results. Studies conducted with African Americans failed to find any harmful physical effects of cigarette smoking on major adulthood depressive disorders [44, 45].

Interestingly, in our study, the likelihood of depressive symptoms in both current smokers and former female smokers was similar. In contrast, current smokers, including smokers who recently started smoking, showed significantly more depressive symptoms than recent former smokers in a study of Flemish women [46]. Furthermore, in one study conducted in Canada, current smoking rather than ever smoking was a major factor in the onset of depression [47]. However, in a study conducted in a Japanese workplace, the prevalence of depression was higher among past female smokers than current female smokers. The researchers suggested that this result might be caused by Japanese women’s lower haemoglobin carbon monoxide binding capacity [48]. It is possible that the total amount of smoking was similar in current and former female smokers in our current study.

Several mechanisms could explain the relationship between smoking and depression [49]. One explanation, which is referred to as ‘self-medication’, assumes that smokers tend to smoke more to deal with depressive symptoms [50]. This hypothesis supports reverse causality in the association between smoking and depression. In our study, there was significant association between SHS and depression in women. This finding is concordant with the result of the study conducted on one birth cohort [13].

It is known that smoking affects dopamine levels in the human brain [51]. In active smokers, nicotine addiction causes dysregulation of the dopaminergic system, which affects vulnerability to depression [52]. An animal study also suggested that there was a certain effect of SHS on the dopamine system of rats [53]. In addition, free radicals generated by smoking tobacco cause oxidative stress, which damages tissues through protein oxidation and lipid peroxidation [54].

It is well known that there is a strong association between smoking and low socio-economic status [55] and that low socio-economic status is also related to exposure to SHS, especially among women. One U.S. study showed that adult women with manual working jobs and shorter educational attainment were more likely to be exposed in SHS in the home [56]. Another study showed that as white and African American women’s socio-economic status decreased, they reported fewer home smoking bans [57]. In our study, although we adjusted for factors such as marital status, education and employment status, it is still possible that these factors, rather than SHS alone, contributed to the onset of depression symptoms.

This study has certain strengths. The study was conducted using a nationwide surveillance database that is representative of the entire South Korean population. In addition, this study focused not only on the smoking status of the participants but also on detailed information about the source of exposure to SHS. This made it possible to explain certain factors that might affect the association between SHS and depressive symptoms.

However, there are several limitations when interpreting the results of this study. First, KNHANES is a cross-sectional survey; therefore the temporal relationship between exposure and outcome is uncertain. In this study, the time sequence between active smoking and depressive symptoms is unclear. Second, exposure to smoking was self-reported. Urine cotinine can be used as an appropriate biomarker to more accurately test environmental smoking exposure. Although urine cotinine was measured in KNHANES, the information was limited to only a small proportion of participants. We did not have a sufficiently large sample size for further analysis. The proportion of women who were confirmed as smokers using urine cotinine analysis was found to be 8.0 % higher than that of women who self-reported as smokers in KNHANES [58]; therefore, we might have underestimated the association between smoking and depressive symptoms. Additionally, exposure to SHS might be underreported, as one study conducted in the U.S. reported a higher prevalence of SHS using serum cotinine than was self-reported [59]. Likewise, it was impossible to calculate the total number of packs smoked per year among the current smokers because of a lack of information in the KNHANES database. Lastly, participants self-reported their depressive symptoms by answering a simple question; a more standardized assessment tool such as the CES-D or the PHQ-9 must be used in further studies.

## Conclusions

In conclusion, women who are current, former or never smokers with exposure to SHS at home have a higher likelihood of experiencing depressive symptoms. Following the enactment of the National Health Promotion Act in Korea in 1995, many public areas such as government buildings, large restaurants, public parks, streets and bus stations were designated as non-smoking areas. However, areas such as pubs and bars have not enforced smoke-free policies. Therefore, this study’s findings that all types of smoking, including involuntary smoking, are associated with depression have implications for these establishments.

## Additional file

Additional file 1:
**Distribution of stress level by smoking status.** (DOCX 16 kb)
