# Supplementary material for: Active smoking and exposure to secondhand smoke and their relationship to depressive symptoms in the Korea national health and nutrition examination survey (KNHANES)
Source: BMC Public Health. 2015 Oct 14;15:1053. doi: 10.1186/s12889-015-2402-1 (PMC4606499; doi:10.1186/s12889-015-2402-1)
Supplement: Additional file 1: — Distribution of stress level by smoking status. (DOCX 16 kb) [file 12889_2015_2402_MOESM1_ESM.docx]

# Additional files

### Additional file 1 – Distribution of stress level by smoking status

| Amount of perceived stress | | Men | | | |  | Women | | | |
| --- | --- | --- | --- | --- | --- | --- | --- | --- | --- | --- |
|  |  | Current smoker | | | | | | | | |
|  |  | SHS exposure | | | | | | | | |
|  |  | No exposure | At home only | At workplace only | At both place |  | No exposure | At home only | At workplace only | At both place |
|  | Very much | 182 (4.2) | 28 (4.8) | 175 (5.0) | 38 (6.8) |  | 70 (9.3) | 48 (13.1) | 29 (10.0) | 19 (11.7) |
|  | Moderate | 824 (20.8) | 122 (23.6) | 908 (26.1) | 167 (30.0) |  | 256 (31.9) | 145 (34.1) | 99 (35.2) | 76 (38.5) |
|  | Somewhat | 2,333 (55.8) | 331 (56.6) | 2,035 (59.9) | 332 (53.8) |  | 370 (44.5) | 175 (44.0) | 132 (46.7) | 87 (43.7) |
|  | Not at all | 990 (19.2) | 130 (14.9) | 341 (9.1) | 63 (9.5) |  | 149 (14.1) | 46 (8.8) | 22 (8.1) | 16 (6.1) |
| Amount of perceived stress | | Former smoker | | | | | | | | |
|  |  | SHS exposure | | | | | | | | |
|  |  | No exposure | At home only | At workplace only | At both place |  | No exposure | At home only | At workplace only | At both place |
|  | Very much | 35 (1.9) | 6 (7.5) | 36 (3.1) | 2 (4.4 ) |  | 16 (4.3) | 3 (4.1) | 4 (8.1) | 2 (10.0) |
|  | Moderate | 249 (14.9) | 12 (21.8) | 219 (21.4) | 13 (26.2) |  | 107 (34.2) | 21 (33.3) | 39 (38.4) | 9 (29.3) |
|  | Somewhat | 1,089 (60.5) | 45 (51.2) | 650 (69.0) | 30 (60.0) |  | 169 (48.9) | 37 (58.3) | 46 (49.5) | 14 (47.9) |
|  | Not at all | 535 (22.7) | 19 (19.5) | 86 (6.6) | 9 (9.4) |  | 61 (12.6) | 6 (4.3) | 4 (4.0) | 3 (12.8) |
| Amount of perceived stress | | Never smoker | | | | | | | | |
|  |  | SHS exposure | | | | | | | | |
|  |  | No exposure | At home only | At workplace only | At both place |  | No exposure | At home only | At workplace only | At both place |
|  | Very much | 49 (2.5) | 3 (2.1) | 40 (3.6) | 5 (3.7) |  | 517 (4.3) | 160 (7.0) | 124 (4.8) | 75 (7.9) |
|  | Moderate | 221 (15.9) | 23 (20.9) | 211 (20.6) | 17 (16.3) |  | 2,518 (21.9) | 583 (26.5) | 699 (30.4) | 290 (29.3) |
|  | Somewhat | 889 (61.0) | 64 (60.0) | 607 (63.5) | 68 (66.3) |  | 6,725 (57.7) | 1,227 (55.4) | 1,331 (56.3) | 544 (54.5) |
|  | Not at all | 373 (20.5) | 22 (17.0) | 116 (12.3) | 17 (13.7) |  | 2,152 (16.1) | 270 (11.1) | 212 (8.4) | 87 (8.3) |
